# Multi‐Omics Analysis and Experimental Validation Identify RAD51 as a Key Biomarker in OSCC

**DOI:** 10.1049/syb2.70048

**Published:** 2025-12-05

**Authors:** Yuanxin Shi, Xie Li, Yueyue Wang, Bin Chen, Guohui Bai

**Affiliations:** ^1^ Key Laboratory of Oral Disease Research School of Stomatology Zunyi Medical University Zunyi China; ^2^ Stomatological Hospital Affliated to Zunyi Medical University Zunyi China

**Keywords:** bioinformatics, biology, oncology

## Abstract

Oral squamous cell carcinoma (OSCC) is an aggressive malignancy associated with high morbidity and mortality. RAD51 recombinase (RAD51), a central DNA repair protein, plays a crucial role in homologous recombination and has been implicated in cancer progression through mechanisms such as genomic instability, chemoresistance and immune modulation. However, its specific function and regulatory mechanisms in OSCC remain incompletely elucidated. We conducted an integrated multiomics analysis including differential expression, single‐cell transcriptomics, prognostic evaluation, functional enrichment and immune infiltration profiling. Experimental validation was performed using siRNA‐mediated RAD51 knockdown in OSCC cell line HSC‐3, followed by functional assays to assess proliferation, migration, invasion, reactive oxygen species (ROS) accumulation and chemosensitivity. RAD51 was significantly overexpressed across multiple cancers, including OSCC, and exhibited high diagnostic accuracy for OSCC (AUC = 0.956). Single‐cell RNA sequencing revealed elevated RAD51 expression in malignant and proliferating T cells, associating it with aggressive phenotypic traits. High RAD51 expression predicted poor prognosis in OSCC and other cancers. Functional analyses indicated its involvement in the Fanconi anaemia pathway, DNA damage repair and cell cycle regulation. Immune infiltration analysis revealed significant negative correlations with multiple immune cell subtypes and tumour microenvironment scores. Experimentally, RAD51 knockdown suppressed malignant behaviours and enhanced ROS production and chemosensitivity in HSC‐3 cells. RAD51 drives OSCC progression by enhancing malignant phenotypes, suppressing immune infiltration, promoting aberrant DNA repair, elevating oxidative stress and promoting therapy resistance. These findings support RAD51's potential as both a prognostic biomarker and a therapeutic target in OSCC.

## Graphical abstract

This study, through multiomics analysis and experimental validation, discovered that RAD51 is significantly overexpressed in oral squamous cell carcinoma (OSCC) and drives tumour progression by promoting malignant phenotypes, suppressing immune infiltration and inducing dysregulated DNA repair. RAD51 can serve as a potential prognostic biomarker and therapeutic target in OSCC, with its high expression closely associated with poor prognosis and chemotherapy resistance.

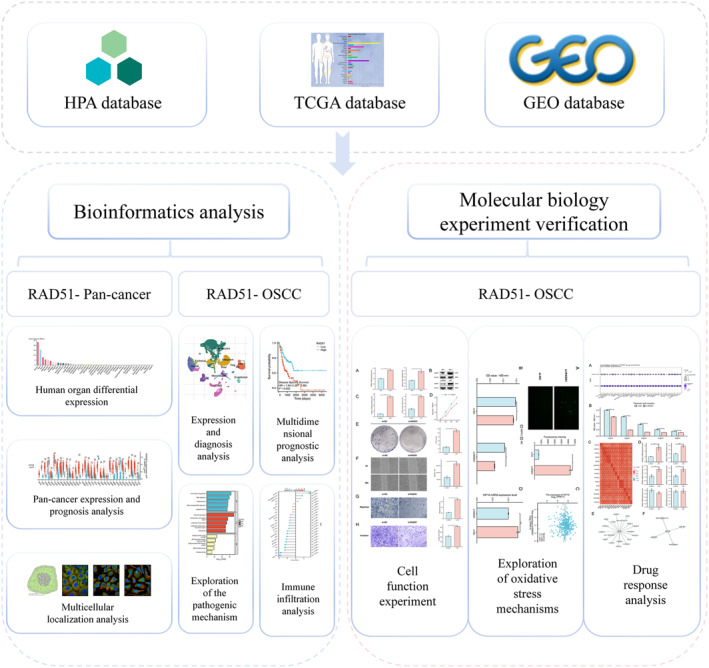

**Abbreviations**
BLCA: bladder urothelial carcinoma
BRCA: breast invasive carcinoma
CESC: cervical squamous cell carcinoma and endocervical adenocarcinoma
CHOL: cholangiocarcinoma
COAD: colon adenocarcinoma
CTRP: Cancer Therapeutics Response Portal
DCs: dendritic cells
DMTU: *N*,*N*′‐Dimethylthiourea
DSS: disease‐specific survival
EMT: epithelial‐mesenchymal transition
ESCA: oesophageal carcinoma
ESCC: oesophageal squamous cell carcinoma
FA: Fanconi anaemia
GBM: glioblastoma multiforme
GDSC: Genomics of Drug Sensitivity in Cancer
GEO: Gene Expression Omnibus
GO: Gene Ontology
GSEA: Gene Set Enrichment Analysis
GSVA: Gene Set Variation Analysis
HNSC: Head and Neck Squamous Cell Carcinoma
HNSCC: Head and Neck Squamous Cell Carcinoma
HPA: Human Protein Atlas
HR: homologous recombination
HRR: homologous recombination repair
iDCs: immature dendritic cells
KEGG: Kyoto Encyclopedia of Genes and Genomes
KIRC: kidney renal clear cell carcinoma
KIRP: kidney renal papillary cell carcinoma
LGG: brain lower grade glioma
LIHC: liver hepatocellular carcinoma
LUAD: lung adenocarcinoma
LUSC: lung squamous cell carcinoma
MESO: malignant mesothelioma
OS: overall survival
OSCC: oral squamous cell carcinoma
PAAD: pancreatic adenocarcinoma
PCPG: pheochromocytoma and paraganglioma
pDCs: plasmacytoid dendritic cells
PFI: progression‐free interval
PRAD: prostate adenocarcinoma
qRT‐PCR: quantitative real‐time polymerase chain reaction
RAD51: RAD51 recombinase
READ: rectum adenocarcinoma
ROC: Receiver Operating Characteristic
ROS: reactive oxygen species
siRNA: small interfering RNA
SKCM: skin cutaneous melanoma
ssGSEA: single‐sample gene set enrichment analysis
STAD: stomach adenocarcinoma
TCGA: The Cancer Genome Atlas
Tfh: T follicular helper
Th1: T helper 1
Th17: T helper 17
THCA: thyroid carcinoma
THYM: thymoma
TME: tumour microenvironment
TPM: transcripts per million
Tregs: regulatory T cells
UCEC: uterine corpus endometrial carcinoma
UVM: uveal melanoma

## 1 Introduction

Oral squamous cell carcinoma (OSCC) represents one of the most prevalent head and neck cancers (HNSCC), accounting for over 90% of all oral malignant tumours [1]. OSCC commonly affects oral structures, including the tongue, gingiva, buccal mucosa and other sites, leading to compromised masticatory and swallowing functions, along with disfigurement and speech impairment. These functional deficits significantly diminish patients' quality of life [2]. Despite modest improvements from current therapeutic modalities, including surgical resection and adjuvant chemoradiotherapy, the prognosis of patients with OSCC remains poor, particularly among those diagnosed with advanced‐stage disease [3]. The 5‐year survival rate of OSCC remains among the lowest of all solid malignancies and has plateaued at approximately 50% over the past three decades [4]. The high mortality rate of OSCC primarily stems from two critical challenges: the absence of reliable early‐diagnostic biomarkers and the deficiency of specific molecular targets for precision therapies to improve prognosis. Consequently, identifying effective prognostic biomarkers for early detection and exploring novel therapeutic targets hold significant clinical importance for management of patients with OSCC [5].

To identify such biomarkers and targets, attention has turned to molecular mechanisms underlying tumour progression, particularly DNA repair pathways. Among these, the homologous recombination (HR) pathway is critical for genomic stability. RAD51 recombinase (RAD51), as a key effector of HR, has thus become a primary candidate for investigation in OSCC. Supporting this direction, analysis of TCGA‐OSCC data shows that high RAD51 expression significantly correlates with poor patient prognosis, and independent clinical studies have further validated RAD51 as a prognostic biomarker in OSCC [6].

RAD51 is a crucial DNA repair protein with a significant pro‐oncogenic role in multiple cancers. Elevated RAD51 expression and homologous recombination activity are associated with increased genomic instability, driven by both spontaneous and therapy‐induced DNA breaks. This instability ultimately promotes chemoresistance, immune dysregulation and poor prognosis across various malignancies [7]. In hepatocellular carcinoma, RAD51 knockdown suppresses cancer cell proliferation, migration and invasion while enhancing apoptosis and DNA damage accumulation [8]. In oesophageal squamous cell carcinoma (ESCC), patients with high RAD51 expression exhibit significantly reduced survival rates; RAD51 depletion attenuates cancer cell viability by inhibiting cell cycle progression and epithelial‐mesenchymal transition (EMT)‐mediated migration/invasion, whereas RAD51 overexpression promotes cancer progression through the p38/Akt/Snail signalling pathway [9]. In prostate cancer, RAD51 upregulation enhances intrinsic chemoresistance via EMT and DNA repair pathways [10]. Furthermore, RAD51 promotes cancer progression through distinct mechanisms in pancreatic cancer [11], cervical squamous cell carcinoma [12], lung adenocarcinoma [13], malignant glioma [14] and invasive ductal breast carcinoma [15].

**FIGURE 1:**
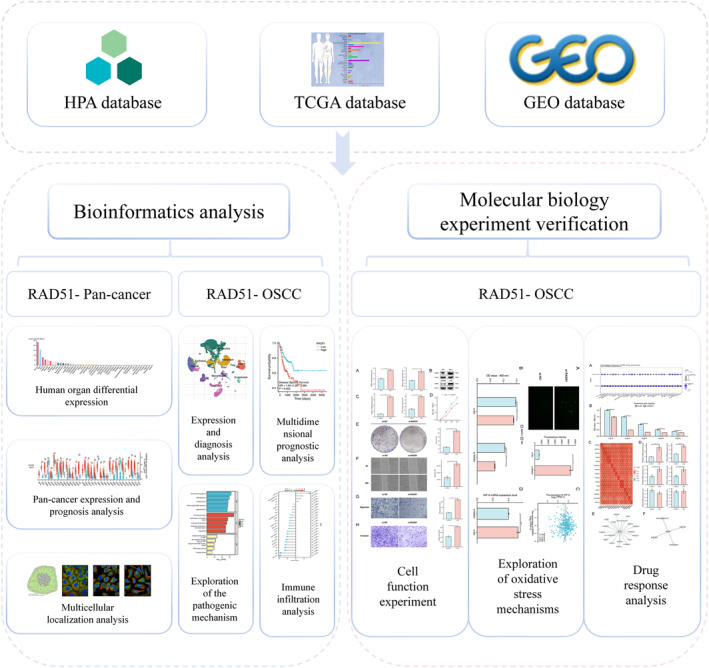
Integrated bioinformatics and experimental workflow.

However, the prognostic significance, functional role, and underlying mechanisms of RAD51 in OSCC remain incompletely understood. Integrative multiomics analysis offers a comprehensive strategy to decipher complex oncogenic mechanisms by harmonising data across multiple biological layers [16, 17]. Recent advances in computational biology, including deep learning approaches for multimodal data integration, have demonstrated the capability to extract biologically meaningful insights from complex datasets [18, 19]. Inspired by these methodological progress, we employed a combined approach of systematic bioinformatics and experimental validation to investigate RAD51 in OSCC. We assessed its expression at transcriptomic and single‐cell levels, prognostic value, functional pathways, coexpression networks and immune infiltration associations. To experimentally validate its functional impact, we selected the HSC‐3 cell line, a model for tongue squamous cell carcinoma, which represents the most aggressive subtype of OSCC. Tongue squamous cell carcinoma carries the worst prognosis among OSCC subtypes, with approximately 30% of early‐stage cases metastasising to cervical lymph nodes [20], making HSC‐3 a relevant model for studying aggressive OSCC. Using siRNA‐mediated knockdown of RAD51 in HSC‐3 cells, we comprehensively evaluated its effects on proliferation, invasion, migration, ROS accumulation and chemoresistance. The overall study design and workflow, outlining steps from data analysis to experimental validation, are summarised in Figure 1. Our findings advance the understanding of RAD51's role in OSCC and may inform the development of novel diagnostic and therapeutic strategies.

## 2 Materials and Methods

### 2.1 Data Collection

Bioinformatics data were primarily sourced from The Cancer Genome Atlas (https://portal.gdc.cancer.gov) (TCGA) [21] database and Gene Expression Omnibus (GEO, https://www.ncbi.nlm.nih.gov/geo/) [22] database. RNA‐sequencing data in TPM format were obtained from public databases including TCGA and GEO. Expression values were transformed using log2 (TPM + 1) to stabilise variance. No specific data filtering was applied unless otherwise stated.

### 2.2 Reagents and Materials

The primary materials utilised in this study comprised: the human immortalised tongue squamous cell carcinoma line HSC‐3(OTWO Biotech, China); normal human oral epithelial cell line HOK (OTWO Biotech, China); total RNA Extraction Kit (Takara, Japan); PrimeScript cDNA Synthesis Kit (Takara Bio, Japan); TB Green Premix Ex Taq II (Tli RNaseH Plus) (Takara, Japan); DMEM medium (Gibco, USA); foetal bovine serum (Thermo Fisher, China); siRNAplus Transfection Reagent (Jinbaoluo, China); anti‐RAD51 antibody (1:10,000, ab133534, Abcam, UK); anti‐β‐actin antibody (1:5,000, AB‐2923704, Proteintech, China); goat antirabbit secondary antibody (1:10,000, AB‐2722564, Proteintech, China); CCK‐8 Cell Proliferation Assay Kit (Beyotime, China); BL1833B Matrigel (Biosharp, China); 4% cell/tissue fixative (Solarbio, China); Transwell chambers with 0.1% crystal violet staining solution (Solarbio, China); cisplatin (Yuanye Bio, China); reactive oxygen species detection reagent (Biosharp, China) and the ROS scavenger *N*,*N*′‐Dimethylthiourea (DMTU) (KKL Med, USA). siRNAs were procured from Genepharma (China), with all mRNA primers synthesised by Sangon Biotech (China).

## 3 Method

### 3.1 Pan‐Cancer Analysis of RAD51 Expression and Prognostic Value

RAD51 mRNA expression in normal tissues (*N* = 13,084) was examined using data from the Human Protein Atlas (HPA, https://www.proteinatlas.org/) [23]. For the pan‐cancer analysis, RNA‐seq data from 33 TCGA cancer projects were downloaded. A grouped comparison of RAD51 expression across these cancer types was performed. Additionally, a matched‐pair analysis was conducted using tumour and adjacent normal samples from the consolidated TCGA‐ALL dataset, with the Wilcoxon signed‐rank test used for statistical evaluation. Immunofluorescence staining images from three HPA cell lines (A‐431, U251 MG and U‐2 OS) were used to demonstrate the subcellular localisation of RAD51 in cancer cells. The prognostic value of RAD51 was assessed using TCGA RNA‐seq data across three endpoints: overall survival (OS), disease‐specific survival (DSS) and progression‐free interval (PFI). Univariate Cox proportional hazards regression analysis (using the survival package) was performed for each cancer type. Results were visualised as a forest plot (ggplot2), and prognostic annotations were sourced from Liu et al. [24].

### 3.2 Expression of RAD51 in OSCC and Its Relationship With Clinical Pathological Characteristics

To analyse RAD51 expression in oral squamous cell carcinoma (OSCC), RNA‐seq data and clinical information from the TCGA Head and Neck Squamous Cell Carcinoma (TCGA‐HNSC) project were retrieved. Samples from nonoral cavity HNSCC sites (e.g., Hypopharynx and Larynx) were excluded, retaining only those from oral cavity subsites (e.g., Oral Tongue and Buccal Mucosa). The final cohort included 32 normal and 330 tumour samples for unpaired analysis. A separate paired analysis was performed on 32 matched tumour‐normal pairs using a paired *t*‐test. The relationship between RAD51 expression and various clinicopathological features (e.g., age and TNM stage) was visualised via scatter plots. Group differences were assessed using one‐way ANOVA with Tukey's HSD post hoc test. Logistic regression models were employed to evaluate the predictive value of RAD51 expression (dichotomised by median) for clinical variables. The diagnostic accuracy of RAD51 was assessed by plotting Receiver Operating Characteristic (ROC) curves using the pROC package (v1.18.0). Furthermore, differential gene expression analysis was conducted in R (v4.2.1) using DESeq2 (v1.36.0) and edgeR (v3.38.2) packages [25, 26], with normal tissues as the reference. DEGs were identified at a threshold of |Log2FC| > 1 and p. adj < 0.05, yielding 5074 genes. The VST‐normalised results were visualised in a volcano plot using ggplot2 (v3.4.4).

### 3.3 Validation of RAD51 Expression Levels in OSCC

To validate the overexpression of RAD51 in OSCC, we analysed three independent datasets from the Gene Expression Omnibus (GEO): GSE23558 [27], GSE74530 [28] and GSE37991 [29]. The CancerSEA database (http://biocc.hrbmu.edu.cn/CancerSEA/) [30] was utilised to investigate the associations between RAD51 and 14 distinct functional states in cancer cells at the single‐cell level. Furthermore, a detailed single‐cell analysis of RAD51 expression in OSCC and HNSCC was performed using the Tumour Immune Single‐Cell Hub 2 (TISCH2, http://tisch.comp‐genomics.org/) portal [31], which integrates data from three GEO datasets (GSE139324 [32], GSE172577 [33] and GSE103322 [34]).

### 3.4 Prognostic Value Analysis of RAD51 in OSCC

To evaluate the prognostic significance of RAD51 in OSCC, Cox proportional hazards regression was performed using the survival (v3.3.1) and rms (v6.3‐0) packages. Variables with *p* < 0.1 in univariate analysis were incorporated into the multivariable Cox model, assessing the impact of gender, age, TNM stage, pathological grade, radiotherapy and RAD51 mRNA expression on overall survival (OS). Forest plots visualised Cox regression results. A prognostic nomogram predicting 1‐, 3‐ and 5‐year survival probabilities was developed (rms v6.3‐0), complemented by a risk score distribution plot quantifying factor contributions. Calibration curves (rms v6.3‐0) assessed model accuracy by comparing predicted versus observed survival at specified timepoints. Kaplan–Meier survival analysis (survminer v0.4.9) with log‐rank test evaluated differences in OS, disease‐specific survival (DSS) and progression‐free interval (PFI) between RAD51 median‐expression stratified groups. All visualisations were generated with ggplot2 (v3.4.4).

### 3.5 Functional Enrichment Analysis via GO, KEGG and GSEA

Spearman correlation analysis was applied to identify genes coexpressed with RAD51 [ENSG00000051180.17] in the TCGA‐OSCC cohort. A heatmap was used to visualise the top 30 positively and negatively correlated genes. All subsequent functional enrichment analyses were performed using data from *Homo sapiens*. The top 300 genes coexpressed with RAD51 were selected for Gene Ontology (GO) [35] and Kyoto Encyclopedia of Genes and Genomes (KEGG) [36] pathway enrichment analyses using the clusterProfiler package (v4.4.4) [37]. Gene set enrichment analysis (GSEA) [38] was performed using the clusterProfiler package on TCGA‐OSCC samples stratified by median RAD51 expression. The reference gene set was c2.cp.all.v2022.1.Hs.symbols.gmt from the MSigDB Collections (https://www.gsea‐msigdb.org/gsea/msigdb/collections.jsp). The org.Hs.eg.db database was used for gene identifier conversion throughout the enrichment analyses.

### 3.6 Immune Infiltration Analysis of RAD51 in OSCC

To assess the relationship between RAD51 expression and immune cell infiltration in OSCC, we employed the ssGSEA algorithm via the GSVA R package (v1.46.0; Hänzelmann et al. [39]) using gene markers for 24 immune cell types from Bindea et al. [40] to calculate immune infiltration scores. Results were visualised through lollipop plots, with association strength quantified by Spearman correlation coefficients and displayed in scatter plots. Statistical comparison of immune cell enrichment between RAD51‐high and RAD51‐low groups was performed using Mann–Whitney *U* tests (stats v4.2.1; car v3.1‐0). Additionally, the ESTIMATE package (v1.0.13; Yoshihara et al. [41]) was applied to examine correlations between RAD51 expression and ImmuneScore, StromalScore and ESTIMATEScore, providing insights into nontumour cellular infiltration based on immune/stromal marker expression profiles. Finally, Spearman analysis evaluated associations between RAD51 expression and 15 immune checkpoints (including TIGIT, PDCD1, TNFRSF4, LAG3 and CTLA4) in the TCGA‐OSCC cohort, visualised through correlation heatmaps. All visualisations were generated with ggplot2 (v3.4.4).

### 3.7 Drug Response Analysis of RAD51

The GSCALite platform (http://bioinfo.life.hust.edu.cn/web/GSCALite/) [42] was employed to analyse integrated mRNA expression data from TCGA and drug sensitivity datasets from the Cancer Therapeutics Response Portal (CTRP; http://portals.broadinstitute.org/ctrp/) [43] and Genomics of Drug Sensitivity in Cancer (GDSC; https://www.cancerrxgene.org/) [44]. Using CTRP data, we examined the relationship between mRNA expression levels of RAD51 and its accessory protein RAD51AP1 with drug sensitivity profiles. Finally, leveraging the GDSC and CTRP databases, we identified compounds exhibiting a statistically significant positive correlation between drug sensitivity and RAD51 expression (cor > 0.1 and fdr < 0.05).

### 3.8 siRNA Interference and qRT‐PCR Analysis

Human oral keratinocytes (HOK) and tongue squamous carcinoma cells (HSC‐3) were maintained in DMEM supplemented with 10% foetal bovine serum under standard culture conditions. Total RNA was extracted from these cell lines, followed by first‐strand cDNA synthesis for subsequent quantitative real‐time polymerase chain reaction (qRT‐PCR) analysis. For each experimental group, a minimum of three independent biological replicates were performed and each biological replicate was assayed in a minimum of three technical replicates during the qRT‐PCR run. Amplification reactions were performed on a Roche LightCycler 96 system using the following protocol: initial denaturation at 95°C for 120 s; 40 cycles of denaturation at 95°C for 20 s and annealing/extension at 56°C for 60 s. Relative quantification was calculated using the 2^ (−ΔΔCt) method. siRNAs targeting RAD51 mRNA sequences were designed and transfected into HSC‐3 cells. Post‐transfection, qRT‐PCR assessed both knockdown efficiency and expression changes of another gene. All primer sequences are provided in Table 1.

**TABLE 1:** The sequence of primers.

Gene	Forward primer (5′‐3′)	Reverse primer (5′‐3′)
si‐RAD51	GACUGGAUCUAUCACGAATT	UUCUGUGAUAGAUCCAGUCTT
si‐NC	UUCUCCGAACGUGUCACGUTT	AGGUGACACGUUCGGAGAATT
RAD51	TGGAGTGGGAGATGCCAAAG	CCTGTAGAGAACCCCAGGGA
GAPDH	CAGGAGGCATTGCTGATGAT	GAAGGCTGGGGCTCATTT
HIF1A	GAACGTCGAAAAGAAAAGTCTCG	CCTTATCAAGATGCGAACTCACA
FANCD2	AAAACGGGAGAGAGTCAGAATCA	ACGCTCACAAGACAAAAGGCA
FANCA	TTTGCTTGAGGTAGAAGGTCCA	CCCGGCTGAGAGAATACCCA
FANCB	ATGAAGGATGGCCTAAGGGTC	ACACACTAACAACTTTGCCAGT
FANCC	TCAAGGTCTTGGGTATGCACC	GCCATTCGCCTTTGAGTGTTAAA
FANCI	GACGAGCTATTGGATGTTGTCA	TTCACGAGTTCTCTGCCTAGT
BLM	GGACCTTGACACCTCTGACAG	GGATTCAGCTCCTGCATACTCA

### 3.9 Western Blotting

Following qRT‐PCR, western blot analysis was performed on HOK cells, HSC‐3 cells and siRNA‐transfected HSC‐3 cells. The experiment was independently repeated at least three times (*n* ≥ 3 biological replicates) to ensure reproducibility. The procedure involved cell collection and lysis using RIPA buffer for total protein extraction. Protein samples were mixed with Laemmli buffer, heat‐denatured and separated by SDS‐PAGE electrophoresis. Proteins were transferred to 0.45 μm PVDF membranes, followed by blocking with 5% skim milk in TBST to reduce nonspecific binding. Membranes were incubated with primary antibodies overnight at 4°C, washed, then probed with HRP‐conjugated secondary antibodies for 1 h at room temperature. Protein detection was achieved through luminol‐based chemiluminescence reagent exposure to X‐ray film, enabling evaluation of RAD51 expression changes among the two cell groups and siRNA transfection efficacy.

### 3.10 Cell Viability Assay (CCK8)

Transfected HSC‐3 cells were seeded in 96‐well plates at a density of 5000 cells per well (*n* = 6 replicates per group). Following respective treatments, 10 μL of CCK‐8 reagent was added to each well at designated timepoints and incubated for 2 h at 37°C. Absorbance was measured at 450 nm using a microplate spectrophotometer to calculate the cell viability for each group.

### 3.11 Clone Formation Assay

Transfected HSC‐3 cells were seeded in 6‐well plates at 500 cells/well and cultured for 14 days. The experiment included six independent biological replicates. The medium was replenished every 2–3 days with colony formation monitored throughout. Following colony development, cells were fixed with 4% paraformaldehyde for 30 min, stained with 0.1% crystal violet for 20 min, air‐dried at room temperature and imaged. Colonies were quantified using ImageJ software.

### 3.12 Wound Healing Assay

Transfected HSC‐3 cells were seeded in 6‐well plates and cultured until approximately 90% confluence. The experiment included four biological replicates, with each replicate comprising three technical replicates (wells). A uniform straight wound was created in the cell monolayer by scratching with a sterile 200 μL pipette tip. After removing detached cells with PBS, fresh medium was added. Wound closure was monitored at 0 and 48 h postscratching using an inverted microscope. The wound area was quantified by tracing the wound edges using the polygon selection tool in ImageJ.

### 3.13 Transwell Migration and Invasion Assay

Transfected HSC‐3 cells were subjected to a 6‐h starvation period, then trypsinized and resuspended in serum‐free medium at a density of 1 × 10^5^ cells/mL. The experiment was independently repeated three times (*n* = 3 biological replicates), with three technical replicates (individual transwell chambers) included for each condition and replicate. For the migration assay, 300 μL of the cell suspension was added to the upper chamber and the lower chamber was filled with 600 μL of medium containing 20% FBS. The invasion assay involved an initial step of coating the upper chamber with 100 μL of diluted Matrigel, allowing it to solidify at 37°C for 1–2 h. The cell suspension was then added, and both assays were conducted for 24–48 h. After incubation, the chambers were washed with PBS, followed by fixation of the cells with 4% fixative for 30 min and staining with 0.1% crystal violet for 20 min. After drying, the chambers were photographed under an inverted microscope and images were saved; the results were analysed using ImageJ software.

### 3.14 Detection of Intracellular Reactive Oxygen Species

Intracellular reactive oxygen species (ROS) levels were detected using an oxidation‐sensitive fluorescent probe, DCFH‐DA (Biosharp, China). Briefly, cells were washed twice with phosphate‐buffered saline (PBS). Subsequently, the samples were incubated with 10 μM DCFH‐DA at 37°C for 20 min according to the manufacturer's instructions. Inside the cells, DCFH‐DA is hydrolysed by nonspecific esterases and is further oxidised by ROS to form the fluorescent compound, 2′,7′‐dichlorofluorescein (DCF). The fluorescence signals of DCF were observed and captured using an inverted fluorescence microscope.

### 3.15 Statistical Analysis

All experimental and bioinformatic data were statistically analysed and visualised using R software (version 4.2.1). The raw data for key experimental assays—including western blot band intensity, colony formation counts, cell migration area in the wound healing assay, the number of migrated/invaded cells in Transwell assays and fluorescence intensity in the ROS detection assay—were initially processed and quantified using ImageJ software (version 1.45 g). This also included data from other in vitro assays (qRT‐PCR and CCK‐8) and bioinformatic analyses (pan‐cancer and OSCC‐specific expression profiles from TCGA and GEO, prognostic survival data and functional enrichment results). The appropriate statistical method was selected based on whether the data met the assumptions of normality and homogeneity of variance, which were checked using the stats (v4.2.1) and car (v3.1‐0) packages. Data visualisation was performed with the ggplot2 package (v3.4.4). The specific statistical tests applied were as follows: For comparisons between two groups: Student's *t*‐test (when data met both normality and homogeneity of variance), Welch's *t*‐test (when data were normal but variances were unequal) or the Wilcoxon rank‐sum test (a nonparametric test used when the normality assumption was violated). For comparisons among multiple groups: One‐way ANOVA (when data met both normality and homogeneity of variance), Welch's one‐way ANOVA (when data were normal but variances were unequal) or the Kruskal–Wallis test (a nonparametric test used when the normality assumption was violated). For paired comparisons: Paired *t*‐test or Wilcoxon signed‐rank test was used as specified in the respective method sections. For correlation analysis: Spearman's rank correlation method was employed. For survival analysis: Kaplan–Meier method with log‐rank test and Cox proportional‐hazards regression model were used. Statistical significance was set at *p < 0.05, **p < 0.01 and ***p < 0.001.

## 4 Results

### 4.1 Expression and Prognostic Significance of RAD51 Across Pan‐Cancer

**FIGURE 2:**
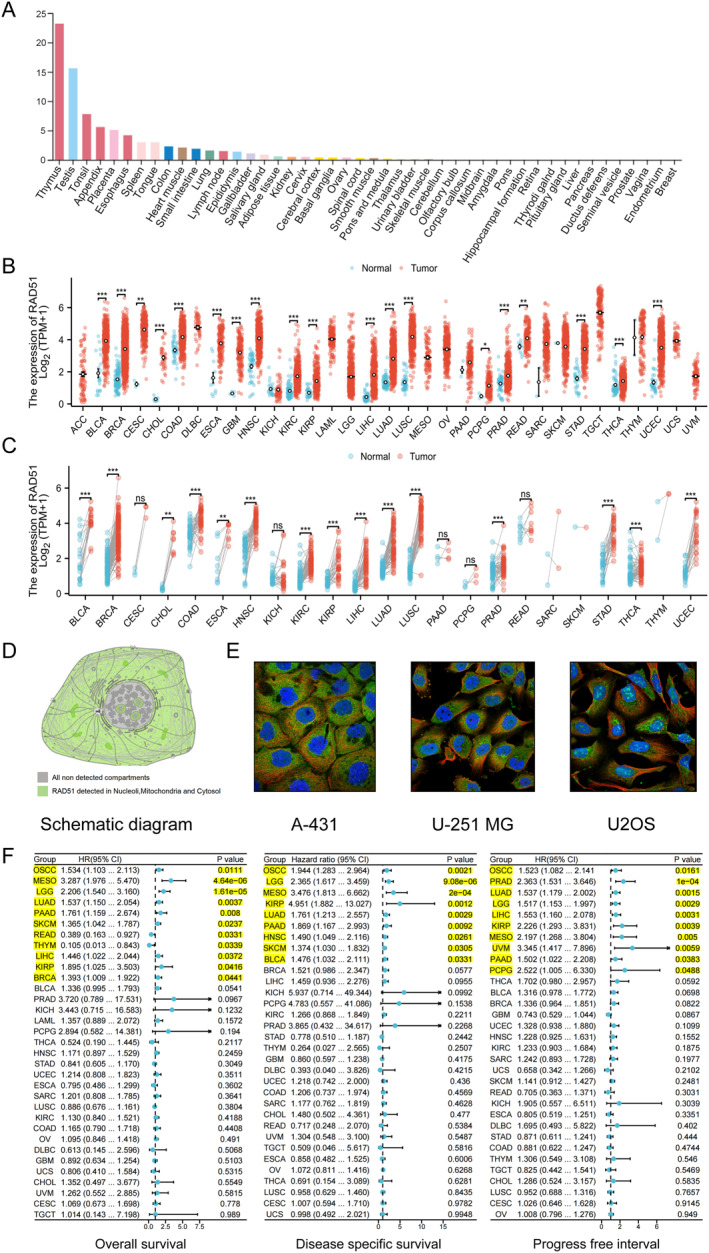
Expression and prognostic significance of RAD51 across pan‐cancer. (A) mRNA expression level of RAD51 in normal tissues based on the HPA dataset. (B) mRNA expression level of RAD51 between tumour and normal tissues from TCGA. (C) mRNA expression level of RAD51 in TCGA tumours and paired adjacent normal tissues. (D, E) Subcellular localisation from the HPA dataset. (F) Forest plot visualisation of pan‐cancer survival analysis for RAD51. (ns, not significant, *p* ≥ 0.05; *p < 0.05; **p < 0.01 and ***p < 0.001).

According to the HPA dataset, RAD51 is highly expressed in the reproductive and digestive systems, with detectable levels also present in the tongue (oral cavity) (Figure 2A). Pan‐cancer expression analysis demonstrated that RAD51 was upregulated in 24 cancer types compared to normal tissues, including bladder urothelial carcinoma (BLCA), breast invasive carcinoma (BRCA), cervical squamous cell carcinoma and endocervical adenocarcinoma (CESC), cholangiocarcinoma (CHOL), colon adenocarcinoma (COAD), oesophageal carcinoma (ESCA), glioblastoma multiforme (GBM), head and neck squamous cell carcinoma (HNSC), kidney renal clear cell carcinoma (KIRC), kidney renal papillary cell carcinoma (KIRP), brain lower grade glioma (LGG), liver hepatocellular carcinoma (LIHC), lung adenocarcinoma (LUAD), lung squamous cell carcinoma (LUSC), pheochromocytoma and paraganglioma (PCPG), prostate adenocarcinoma (PRAD), rectum adenocarcinoma (READ), stomach adenocarcinoma (STAD), thyroid carcinoma (THCA) and uterine corpus endometrial carcinoma (UCEC) (Figure 2B and Table S1). Furthermore, in paired comparisons of tumour and adjacent normal tissues, RAD51 was upregulated in 15 cancer types: BLCA, BRCA, CHOL, COAD, ESCA, HNSC, KIRC, KIRP, LIHC, LUAD, LUSC, PRAD, STAD, THCA and UCEC (Figure 2C and Table S2). In three human cancer cell lines—human epidermoid carcinoma (skin‐derived) A‐431, human glioblastoma U‐251 MG and human osteosarcoma U‐2OS—we observed that RAD51 subcellular localisation was primarily in the nucleoli, mitochondria and cytosol (Figure 2D,E). Pan‐cancer survival analysis of RAD51 revealed that its overall survival (OS) analysis predicted poor prognosis in seven cancers: oral squamous cell carcinoma (OSCC), malignant mesothelioma (MESO), LGG, LUAD, LIHC, KIRP and BRCA; additionally, it predicted favourable prognosis in READ and thymoma (THYM) with statistical significance (*p* < 0.05). RAD51's pan‐cancer disease‐specific survival (DSS) analysis predicted poor prognosis in nine cancers: OSCC, MESO, LGG, KIRP, LUAD, pancreatic adenocarcinoma (PAAD), HNSC, skin cutaneous melanoma (SKCM) and BLCA, with statistical significance (*p* < 0.05). Finally, RAD51's pan‐cancer progression‐free interval (PFI) analysis predicted poor prognosis in 10 cancers: OSCC, PRAD, LUAD, LGG, LIHC, KIRP, MESO, uveal melanoma (UVM), PAAD and PCPG, with statistical significance (*p* < 0.05) (Figure 2F). Therefore, our results indicate that RAD51 expression is upregulated in the majority of cancers. Notably, the pan‐cancer DSS and PFI survival analyses of RAD51 uniformly indicated poor prognosis, whereas only the OS analysis suggested a favourable prognosis in two cancer types. These findings highlight RAD51 as a highly promising therapeutic target in human cancers.

### 4.2 RAD51 Expression in OSCC and Correlation With Clinical Pathological Characteristics

**FIGURE 3:**
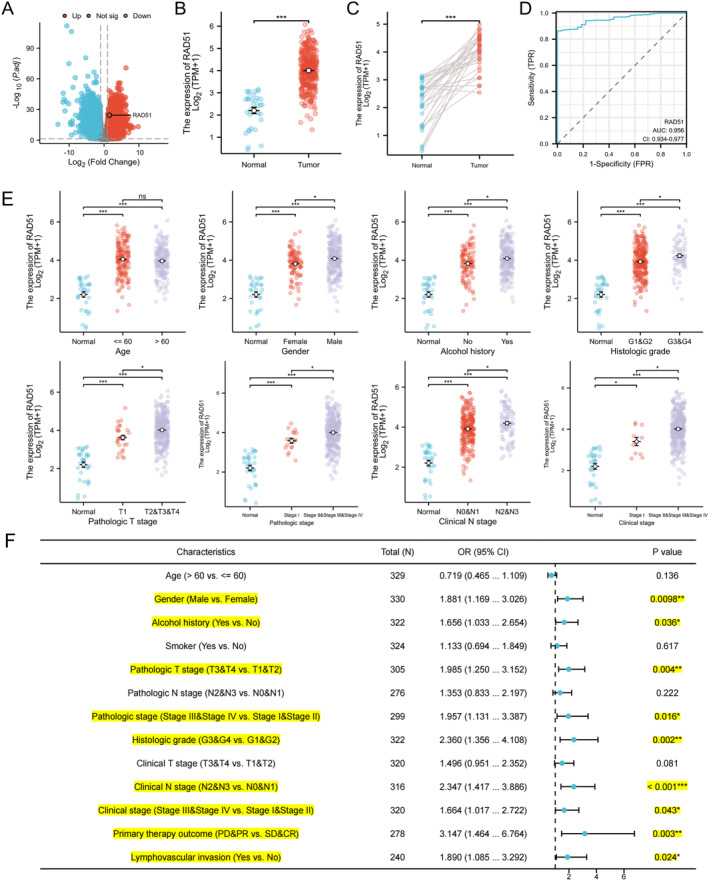
RAD51 expression in OSCC and correlation with clinical pathological characteristics. (A) The volcano plot illustrating all up‐regulated and down‐regulated differentially expressed genes (DEGs) of TCGA‐OSCC. (B) Scatter plot showing the difference in RAD51 expression between OSCC tumour tissue and normal tissue (normal: *n* = 32, 2.2045 ± 0.80028; tumour: *n* = 330, 4.0019 ± 0.7954 and *p* < 0.001). (C) Paired sample comparison of RAD51 expression in OSCC tumour tissue and paired normal tissue (paired normal: *n* = 32, 2.2045 ± 0.80028; tumour: *n* = 32, 4.0122 ± 0.64534 and *p* < 0.001). (D) Receiver operating characteristic (ROC) curve representing the diagnostic potential of RAD51 in OSCC. (E) Scatter plot depicting the association between RAD51 mRNA expression and various clinical variables in patients with OSCC. (F) Forest plot of the single‐gene logistic model analysis for the association between RAD51 expression level and various clinical variables in TCGA‐OSCC. (ns, not significant, *p* ≥ 0.05; *p < 0.05; **p < 0.01 and ***p < 0.001).

Differential analysis of the TCGA‐OSCC dataset revealed significant overexpression of RAD51 in tumour tissues compared to normal controls as illustrated by the volcano plot (Figure 3A). Additionally, analysis of the TCGA‐OSCC cohort confirmed that RAD51 was significantly upregulated in OSCC tissues compared to normal or paired tissues (Figure 3B,C). The diagnostic ROC curve analysis demonstrated a high diagnostic value for OSCC, with an AUC of 0.956 (95% CI: 0.934–0.977) (Figure 3D). One‐way ANOVA analysis indicated that RAD51 expression was significantly associated with several clinicopathological features, including patient gender, alcohol history, histological grade, pathological T‐stage, pathological stage, clinical N‐stage and clinical stage (*p* < 0.05) (Figure 3E and Table S3). Specifically, higher RAD51 expression was observed in males, patients with alcohol history and those with higher histological grade, pathological T‐stage, pathological stage, clinical N‐stage and clinical stage. Single‐gene logistic regression analysis, using RAD51 as the independent variable, revealed that higher RAD51 expression was associated with male gender (OR = 1.881 and *p* < 0.01), alcohol history (OR = 1.656 and *p* < 0.05), higher pathological T‐stage (OR = 1.985 and *p* < 0.01), pathological stage (OR = 1.957 and *p* < 0.05), histological grade (OR = 2.360 and *p* < 0.01), clinical N‐stage (OR = 2.347 and *p* < 0.001) and clinical stage (OR = 1.664 and *p* < 0.05). Additionally, patients with lymph node invasion exhibited higher RAD51 expression (OR = 1.890 and *p* < 0.001). Patients with high RAD51 expression also showed a poorer treatment response (PD & PR vs. SD & CR: OR = 3.147 and *p* < 0.01) (Figure 3F). Collectively, these findings demonstrate consistent upregulation of RAD51 in OSCC across multiple datasets and methodologies (TCGA, GEO and IHC), its high diagnostic value and significant associations with adverse clinicopathological features and poorer treatment response. Thus, RAD51 emerges as a critical biomarker linked to OSCC progression and prognosis.

### 4.3 Validation of RAD51 Expression Levels in OSCC Using External Datasets

**FIGURE 4:**
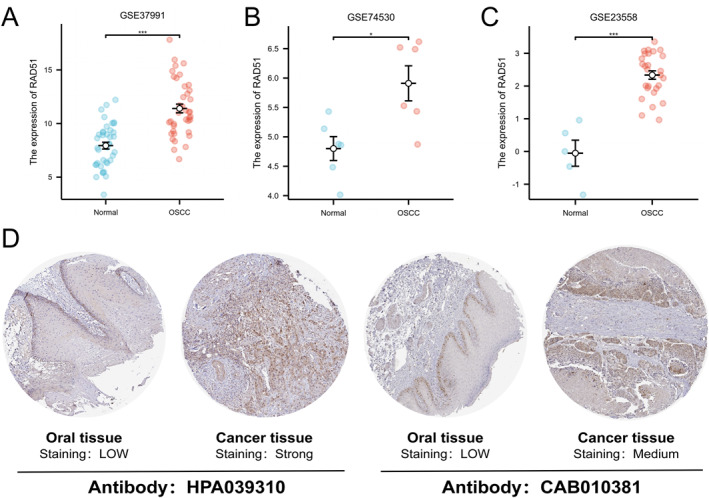
Validation of RAD51 expression in GEO and HPA databases. (A–C)Validation of RAD51 expression difference between OSCC tumour tissue and normal tissue using three independent GEO datasets: GSE37991 (normal: *n* = 40, 7.9395 ± 2.0178 and tumour: *n* = 40, 11.406 ± 2.5725), GSE74530 (normal: *n* = 6, 4.8008 ± 0.49719 and tumour: *n* = 6, 5.91 ± 0.72928) and GSE23558 (normal: *n* = 5, −0.052192 ± 0.89106 and tumour: *n* = 27, 2.336 ± 0.66967). (D) Differential RAD51 protein expression in immunohistochemistry (IHC) sections of normal oral epithelial tissue and OSCC tumour tissue from the HPA database. (ns, not significant, *p* ≥ 0.05; *p < 0.05; **p < 0.01 and ***p < 0.001).

To begin, we validated the mRNA expression of RAD51 in oral squamous cell carcinoma (OSCC) compared to normal tissues using three independent datasets from the GEO database: GSE37991, GSE74530 and GSE23558 (Figure 4A–C) (*p* < 0.05). All three datasets consistently demonstrated significantly higher RAD51 expression in OSCC tissues relative to normal oral epithelium. Immunohistochemistry (IHC) sections from the HPA database showed that detection using two RAD51 antibodies, HPA039310 and CAB010381, revealed higher RAD51 expression in OSCC tissues compared to normal oral tissues (Figure 4D). Datasets from both the GEO and HPA databases further confirmed the high expression of RAD51 at both the mRNA and protein levels in OSCC, thereby robustly supporting the reliability of our previous findings.

**FIGURE 5:**
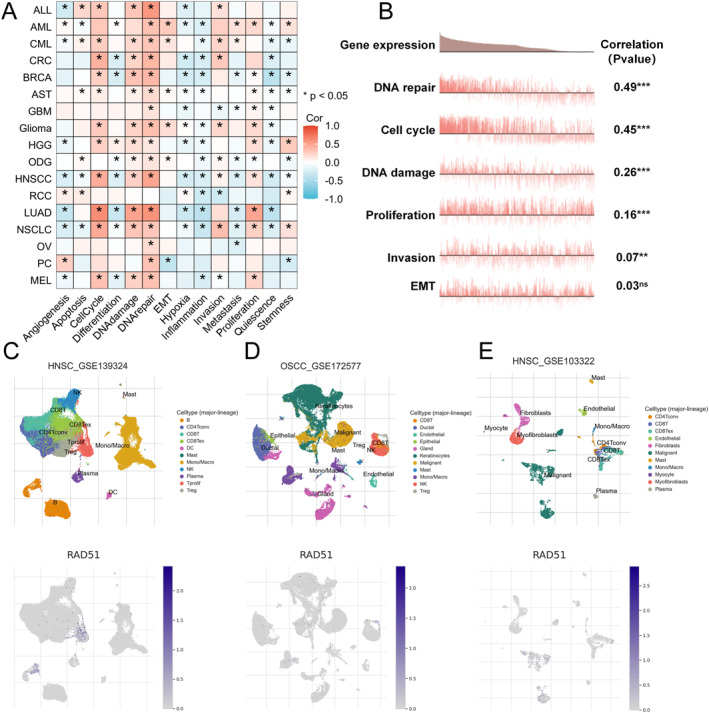
RAD51 expression at single‐cell level in OSCC. (A) The relationship between RAD51 expression and different functional states in tumours was explored by CancerSEA. (B) Correlation analysis between functional status and RAD51 expression in HNSCC. (C–E) RAD51 expression profiles at single‐cell resolution of RAD51 in three single‐cell datasets. (ns, not significant, *p* ≥ 0.05; *p < 0.05; **p < 0.01 and ***p < 0.001).

Single‐cell sequencing is a powerful technology that enables the sequencing and study of genomes, transcriptomes and epigenomes at the individual cell level [45]. We compared and analysed single‐cell sequencing data from the CancerSEA database for RAD51 and its strongly correlated genes, including RAD51B, RAD51C, RAD51D, XRCC2, XRCC3 and RAD51AP1 [46]. The results revealed that RAD51 and its closely associated genes were significantly positively correlated with malignant phenotypes in tumours, including cell proliferation, cell cycle progression, DNA damage repair and invasion (*p* < 0.05) (Figure 5A,B and Table S4). Subsequently, using more precise single‐cell sequencing data from the TISCH2 database, we demonstrated that RAD51 is expressed across multiple cell clusters in three OSCC‐related datasets: HNSCC_GSE139324, OSCC_GSE172577 and HNSCC_GSE103322. Notably, RAD51 expression was particularly elevated in Tprolif cells (Figure 5C and Figure S1A), Treg cells (Figure 5D and Figure S1B) and malignant cells (Figure 5E and Figure S1C). The high expression of RAD51 in malignant cell clusters, along with its correlation with multiple aggressive phenotypes, suggests that it may promote tumour progression primarily by regulating malignant behaviours in cancer cells. Furthermore, the elevated expression of RAD51 in Tprolif cells and Treg cells implies a potential regulatory role for RAD51 in the tumour microenvironment (TME) of OSCC.

### 4.4 Prognostic Value of RAD51 in OSCC

**FIGURE 6:**
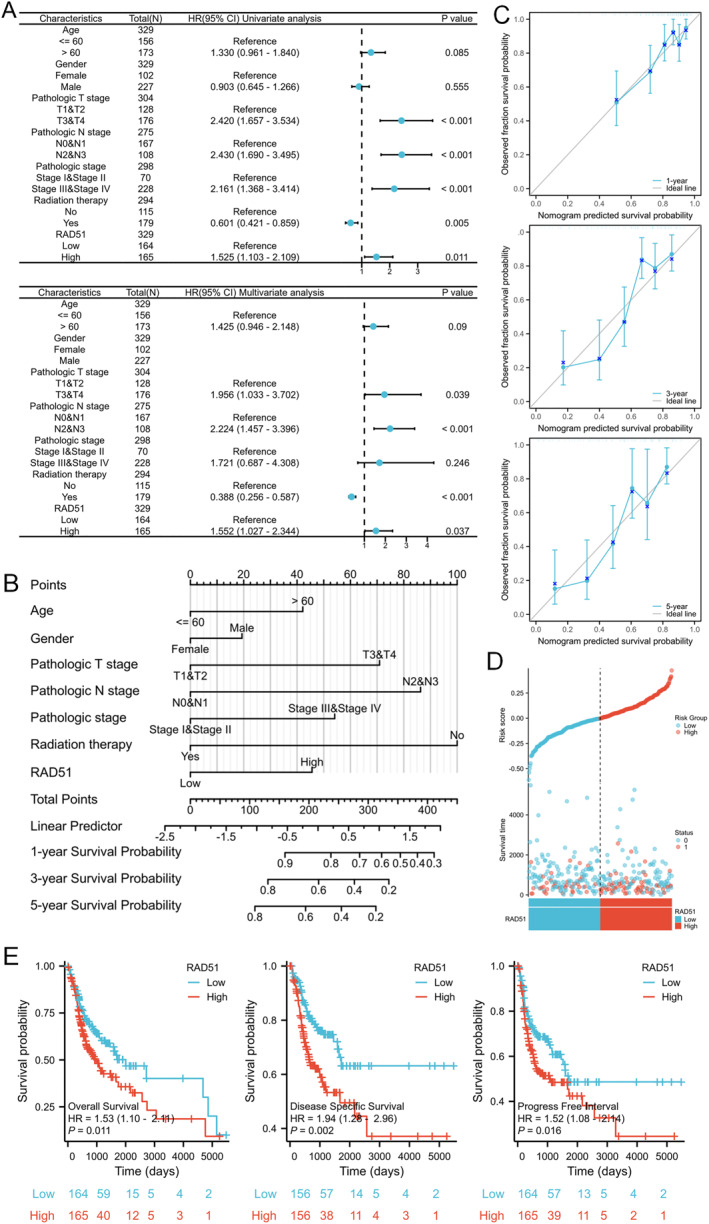
Prognostic value of RAD51 in OSCC. (A) Univariate Cox regression analysis and multivariate Cox regression analysis of clinical pathological characteristics in patients with OSCC; (B) prognostic nomogram based on clinical variables and RAD51 expression to predict overall survival; (C) calibration curves validating the nomogram's predictive accuracy for patients with OSCC survival of 1‐, 3‐ and 5‐year; (D) risk factor plot displaying the prognostic model's risk score and stratification; (E) Kaplan–Meier survival curves comparing the overall survival (OS), disease‐specific survival (DSS) and progression‐free interval (PFI) for patients with different levels of RAD51 expression.

Univariate Cox regression analysis identified T3‐4 stage (*p* < 0.001 and HR = 2.420 [1.657–3.534]), N2‐3 stage (*p* < 0.001 and HR = 2.430 [1.690–3.490]), pathological stage III + IV (*p* < 0.001 and HR = 2.161 [1.368–3.414]) and high RAD51 expression (*p* = 0.011 and HR = 1.525 [1.103–2.109]) as prognostic risk factors for OSCC, whereas radiotherapy (*p* = 0.005 and HR = 0.601 [0.421–0.859]) was a protective factor. Multivariate Cox regression analysis further verified T3 and T4 stage (*p* = 0.039 and HR = 1.956 [1.033–3.702]), N2 and N3 stage (*p* < 0.001 and HR = 2.224 [1.457–3.396]) and high RAD51 expression (*p* = 0.037 and HR = 1.552 [1.027–2.344]) as independent prognostic risk factors for OSCC, and radiotherapy (*p* < 0.001 and HR = 0.388 [0.256–0.587]) as an independent prognostic protective factor (Figure 6A). A nomogram was constructed based on clinical characteristics and RAD51 expression level to accurately predict the 1‐year, 3‐year and 5‐year survival rates of patients with OSCC (Figure 6B). The calibration curve also demonstrated good agreement between predicted and actual survival rates for patients with OSCC at 1, 3 and 5 years (Figure 6C). The risk factor plot depicted the correlation between RAD51 expression and survival outcomes, showing that higher RAD51 expression was associated with an increased risk of disease progression and metastasis (Figure 6D). Patients with elevated RAD51 expression exhibited significantly shorter OS, DSS and PFI (*p* < 0.05) (Figure 6E). Consequently, RAD51 constitutes a high‐priority molecular target for therapeutic intervention and a stratification biomarker with significant prognostic utility in OSCC clinical management.

### 4.5 Pathway and Functional Enrichment Analysis Using GO, KEGG and GSEA

**FIGURE 7:**
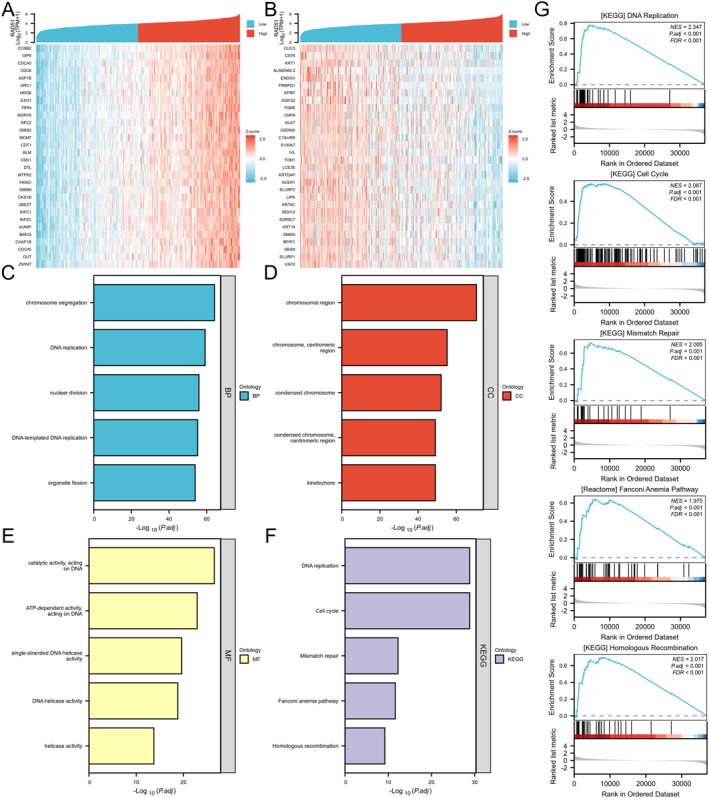
Pathway and functional enrichment analysis using GO, KEGG and GSEA. (A, B) Coexpression heatmap of the top 30 genes positively and negatively correlated with RAD51 in OSCC. (C–E) GO enrichment analysis for the top 300 positively coexpressed genes with RAD51 in OSCC. (F) KEGG enrichment analysis for the top 300 positively coexpressed genes with RAD51 in OSCC. (G) GSEA analysis for patients with OSCC cohorts stratified by RAD51 expression levels.

We performed bulk correlation analysis using the Spearman method between RAD51 and all other molecules in OSCC, obtaining correlation coefficients and *p*‐values. The top 30 genes positively and negatively correlated with RAD51 (adjusted *p*‐value and padj < 0.05) were visualised in a coexpression heatmap (Figure 7A,B and Table S5). Subsequently, GO enrichment analysis revealed that RAD51 and its coexpressed genes were significantly enriched in biological processes (BP) including DNA‐templated DNA replication, DNA replication, chromosome segregation, nuclear division and organelle fission (Figure 7C). In the cellular component (CC) category, enrichment was observed at the kinetochore, condensed chromosome centromeric region, condensed chromosome, centromeric region of the chromosome and chromosomal region (Figure 7D). For molecular function (MF), enrichment occurred in ATP‐dependent activity acting on DNA, catalytic activity acting on DNA, DNA helicase activity, single‐stranded DNA helicase activity and helicase activity (Figure 7E and Table S6). Subsequent KEGG enrichment analysis further indicated enrichment in pathways such as DNA replication, cell cycle, mismatch repair, Fanconi anaemia pathway and homologous recombination (Figure 7F and Table S7). GSEA performed on the TCGA‐OSCC cohort, stratified by RAD51 expression, demonstrated significant enrichment of these KEGG pathways in samples with high RAD51 expression (Table S8). Activation of these pathways suggests that RAD51 may promote cancer cell proliferation and survival by driving the cell cycle, whereas concurrently participating in DNA repair mechanisms—including mismatch repair, the Fanconi anaemia pathway and homologous recombination—to maintain genomic stability and foster chemotherapy resistance. Collectively, these findings indicate that RAD51 likely plays a pivotal role in promoting the malignant progression of OSCC (Figure 7G).

### 4.6 Correlation of RAD51 Expression With Immune Cell Infiltration and Checkpoint

**FIGURE 8:**
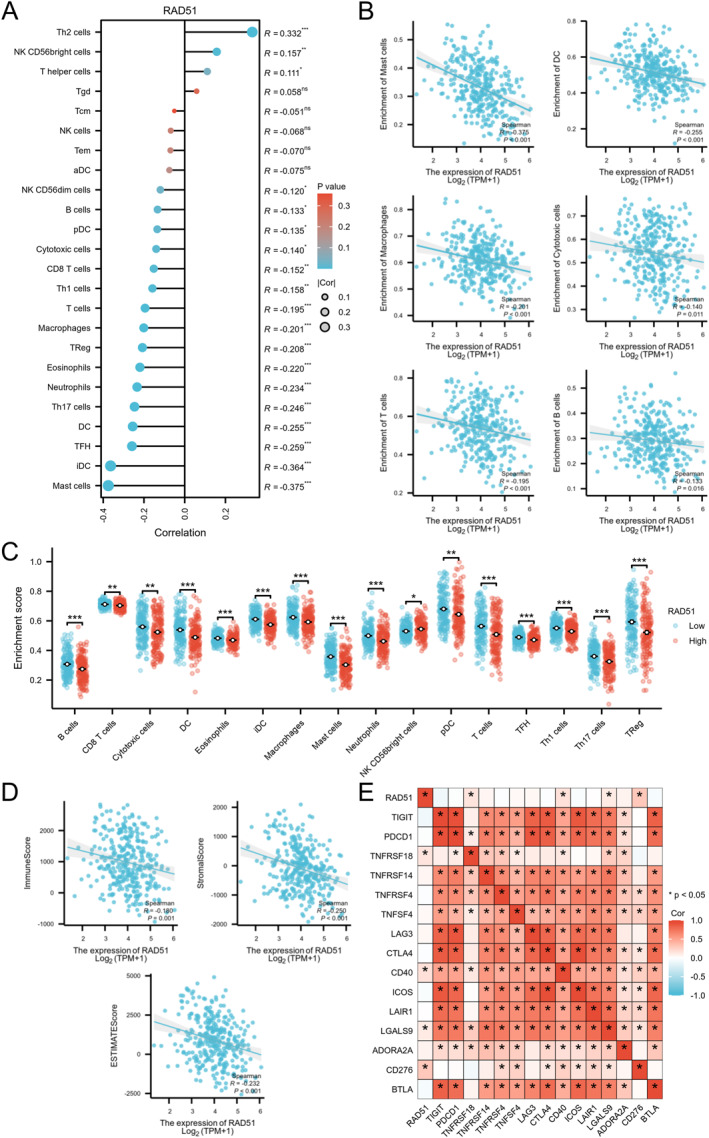
Correlation of RAD51 expression with immune cell infiltration and checkpoint. (A) Lollipop plot depicting correlations between RAD51 expression and infiltration levels of 24 immune cell types. (B) Scatter plots showing associations between RAD51 expression and specific immune cell subtypes. (C) Dot plot comparing immune infiltration scores in high‐ and low‐RAD51 expression groups. (D) Scatter plots illustrating correlations of RAD51 with ImmuneScore, StromalScore and ESTIMATEScore. (E) Heatmap of correlations between RAD51 and 15 common immune checkpoint‐related genes. (ns, not significant, *p* ≥ 0.05; *p < 0.05; **p < 0.01 and ***p < 0.001).

The lollipop plot depicted significant negative correlations (*p* < 0.05) between RAD51 expression and 16 out of 24 immune cell subtypes in OSCC, with only a minority showing positive correlations (Figure 8A). Scatter plots detailed negative correlations with specific immune cells, including mast cells (*R* = −0.374 and *p* < 0.001), dendritic cells (DCs) (*R* = −0.255 and *p* < 0.001), macrophages (*R* = −0.201 and *p* < 0.001), cytotoxic cells (*R* = −0.140 and *p* = 0.011), T cells (*R* = −0.195 and *p* < 0.001) and B cells (*R* = −0.133 and *p* = 0.016) (Figure 8B). Further analysis indicated that RAD51 expression influenced the infiltration of 16 immune cell types—B cells, CD8^+^ T cells, cytotoxic cells, DCs, eosinophils, immature dendritic cells (iDCs), macrophages, mast cells, neutrophils, NK CD56bright cells, plasmacytoid dendritic cells (pDCs), T cells, T follicular helper (Tfh) cells, T helper 1 (Th1) cells, T helper 17 (Th17) cells and regulatory T cells (Tregs)—where the infiltration proportion was significantly reduced in the high‐RAD51 expression group (*p* < 0.05) (Figure 8C). The ESTIMATE algorithm revealed negative correlations between RAD51 expression and ImmuneScore, StromalScore and ESTIMATEScore, suggesting its potential suppressive effect on the immune landscape and tumour microenvironment in OSCC (Figure 8D). Additionally, RAD51 showed significant positive correlations (*p* < 0.05) with immune checkpoint genes TNFRSF18, CD40, CD276 and LGALS9 (Figure 8E). Collectively, RAD51 orchestrates an immunosuppressive landscape in OSCC, characterised by broad negative correlations with immune cell infiltration (16/24 subtypes) and suppressed tumour microenvironment scores. Its positive associations with immune checkpoint genes (TNFRSF18, CD40, CD276 LGALS9) may suggest a potential role in immune evasion. This positions RAD51 as a pivotal regulator of tumour‐immune interactions and a promising immunomodulatory target.

### 4.7 Verification of RAD51 Expression and Its Impact on Biological Behaviour of OSCC

**FIGURE 9:**
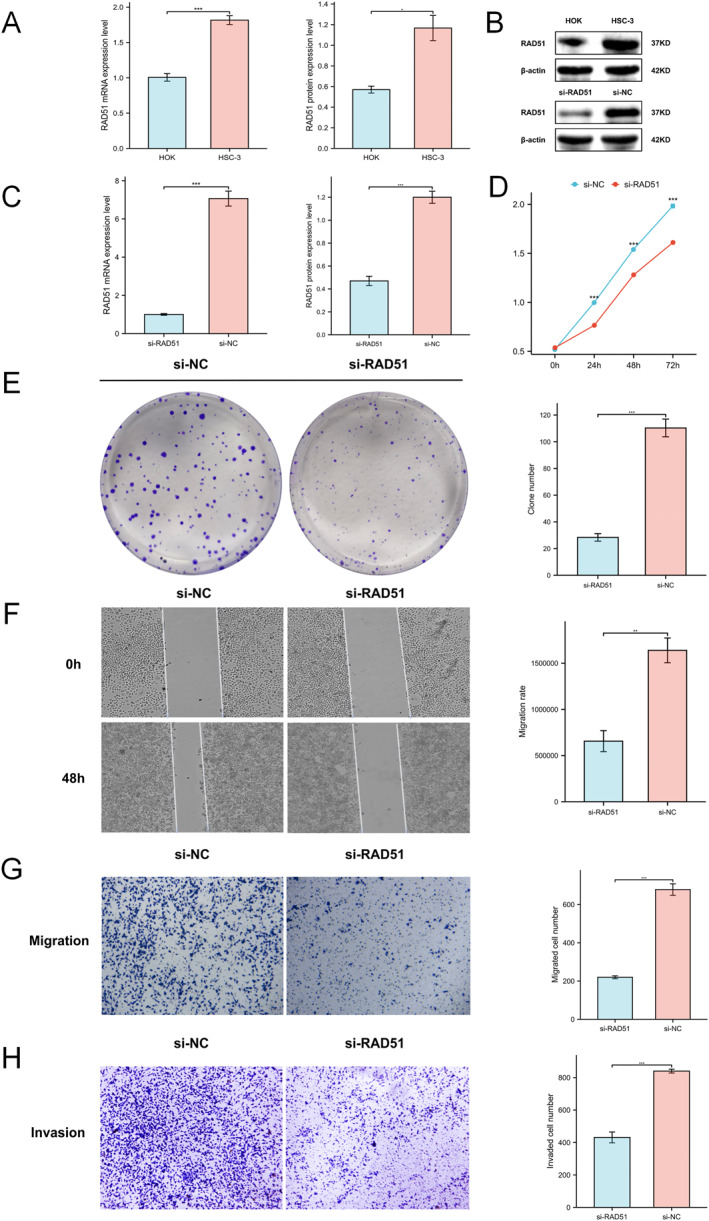
Verification of RAD51 expression and its impact on biological behaviour of OSCC. (A) Quantitative expression of RAD51 mRNA (HOK: *n* = 5, 1.0059 ± 0.12028; HSC‐3: *n* = 5, 1.8168 ± 0.13972 and *p* < 0.001) and protein (HOK: *n* = 4, 0.57 ± 0.06733; HSC‐3: *n* = 4, 1.1675 ± 0.24418 and *p* < 0.05) HOK cells and HSC‐3 cells. (B) Western blot analysis of RAD51 in HOK and HSC‐3 cells. (C) Assessment of RAD51 knockdown efficiency by siRNA using quantitative real‐time PCR (qRT‐PCR) (si‐RAD51: *n* = 3, 1.002 ± 0.077814; si‐NC: *n* = 3, 7.0647 ± 0.67688 and *p* < 0.001) and western blot (si‐RAD51: *n* = 3,0.47 ± 0.07; si‐NC: *n* = 3, 1.2 ± 0.091652 and *p* < 0.001). (D) CCK‐8 assay for cell proliferation evaluation. (E) Colony formation assay assessing proliferative/clonogenic capacity (si‐RAD51: *n* = 6, 28.333 ± 6.9186; si‐NC: *n* = 6, 110.33 ± 16.281 and *p* < 0.001). (F) Wound healing assay determining cell migratory ability (si‐RAD51: *n* = 4, 6.563e+05 ± 2.266e+05; si‐NC: *n* = 4, 1.64e+06 ± 2.679e+05 and *p* < 0.001). (G, H) Transwell migration (si‐RAD51: *n* = 5, 219.8 ± 17.456; si‐NC: *n* = 5, 678.2 ± 67.132 and *p* < 0.001) and invasion (si‐RAD51: *n* = 4, 431.25 ± 67.089; si‐NC: *n* = 4, 840.25 ± 23.768 and *p* < 0.001) assays evaluating cell migration and invasion capacities respectively. (ns, not significant, *p* ≥ 0.05; *p < 0.05; **p < 0.01 and ***p < 0.001).

Compared to the normal human oral keratinocyte cell line HOK, both qRT‐PCR and western blotting assays demonstrated significantly elevated RAD51 mRNA and protein levels in oral squamous cell carcinoma (OSCC) cell line HSC‐3 (Figure 9A,B). Subsequent knockdown experiments in HSC‐3 cells confirmed that small interfering RNA (siRNA) effectively reduced RAD51 expression as validated by qRT‐PCR and western blotting (Figure 9B,C). CCK‐8 assays revealed significantly impaired proliferative capacity in RAD51‐knockdown HSC‐3 cells (*p* < 0.001) (Figure 9D). Similarly, colony formation assays showed markedly reduced proliferative/clonogenic potential (*p* < 0.001) (Figure 9E). Wound healing assays indicated substantially decreased migratory ability (*p* < 0.01) (Figure 9F). Transwell migration and invasion assays further substantiated RAD51's role in HSC‐3 cell motility and invasiveness, with significantly fewer migrating and invading cells postknockdown (*p* < 0.001) (Figure 9G,H). These results underscore RAD51's critical function in promoting proliferation, migration and invasion in OSCC HSC‐3 cells.

### 4.8 RAD51 Depletion Impairs OSCC Proliferation via ROS Accumulation

**FIGURE 10:**
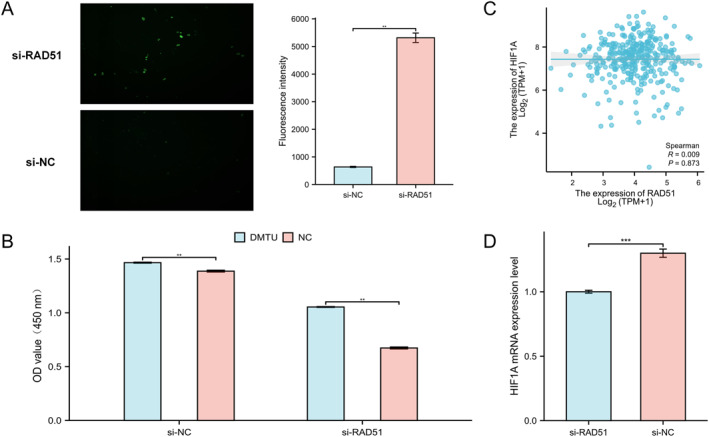
RAD51 depletion impairs OSCC proliferation via ROS accumulation. (A) ROS production in HSC‐3 cells post‐RAD51 knockdown (si‐RAD51: *n* = 3, 636.67 ± 39.068; si‐NC: *n* = 3, 5315.3 ± 297.21 and *p* < 0.01). (B) Cell proliferation assessed by CCK‐8 assay in si‐NC and si‐RAD51 groups treated with DMTU. (C) Correlation analysis of RAD51 and HIF1A expression in TCGA‐OSCC. (D) HIF1A mRNA expression levels following RAD51 knockdown (si‐RAD51: *n* = 6, 1.0003 ± 0.027824; si‐NC: *n* = 6, 1.2996 ± 0.07821 and *p* < 0.001). (ns, not significant, *p* ≥ 0.05; *p < 0.05; **p < 0.01 and ***p < 0.001).

Previous studies have indicated that increased intracellular ROS levels can lead to activation of the DNA damage and repair pathway [47]. However, the impact of DNA damage repair protein RAD51 on oxidative stress in OSCC remains unclear. Using fluorescence microscopy, we observed significantly elevated ROS production in RAD51‐knockdown HSC‐3 cells (*p* < 0.01) (Figure 10A). Subsequent application of the ROS scavenger *N*,*N*′‐Dimethylthiourea (DMTU) to HSC‐3 cells, followed by CCK‐8 viability assessment at 48h, revealed that while DMTU enhanced cell viability in both si‐NC and si‐RAD51 groups compared to NC controls, this prosurvival effect was more pronounced in the si‐RAD51 group (Figure 10B). These findings suggest that RAD51 knockdown may inhibit HSC‐3 cell proliferation by promoting ROS accumulation. Given that intracellular ROS accumulation modulates HIF1A [48], we evaluated RAD51's influence on HIF1A. Although Spearman analysis of TCGA‐OSCC data showed no significant correlation between RAD51 and HIF1A expression (Figure 10C), qRT‐PCR demonstrated that RAD51 knockdown significantly suppressed HIF1A mRNA levels (Figure 10D), further strengthening the reliability of our results. Collectively, RAD51 knockdown likely inhibits HSC‐3 cell proliferation by inducing ROS accumulation.

### 4.9 RAD51 Depletion Impairs Chemoresistance in OSCC

**FIGURE 11:**
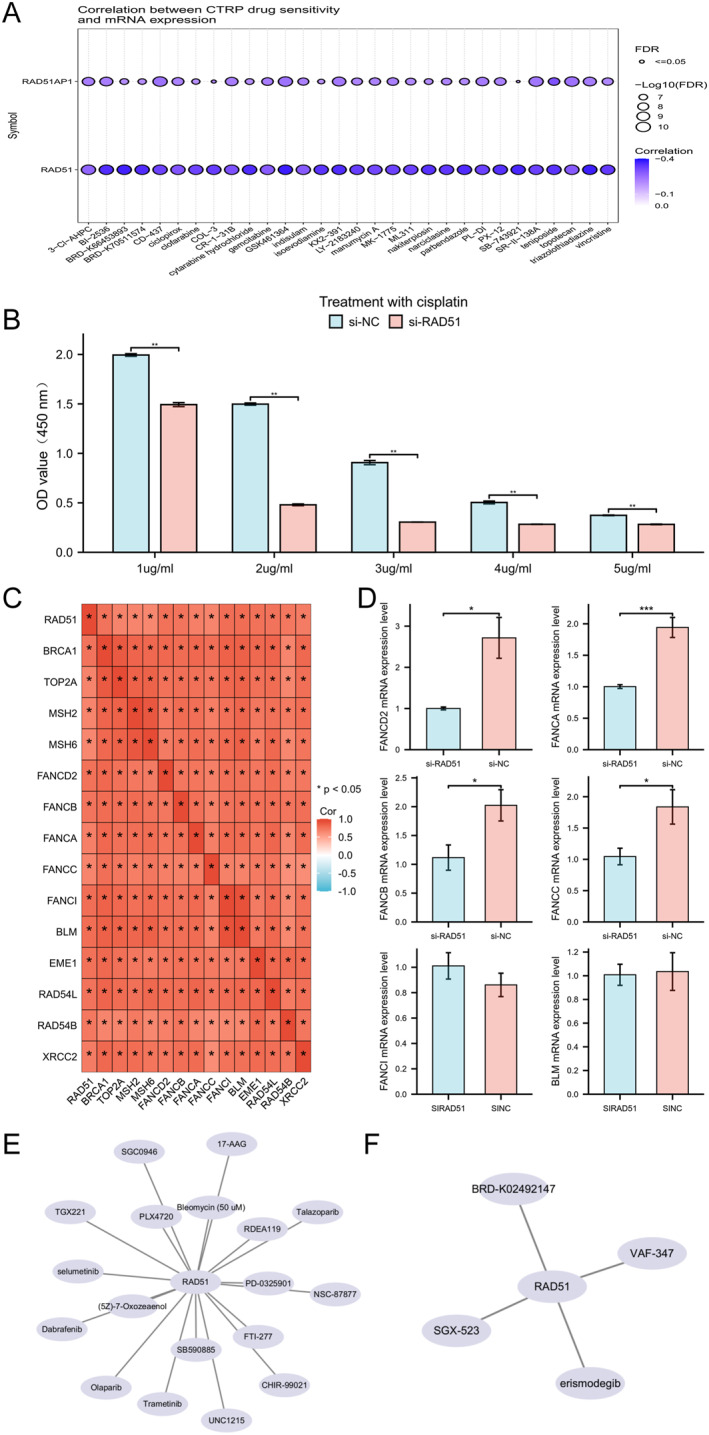
RAD51 depletion impairs chemoresistance and suppresses Fanconi anaemia pathway in OSCC. (A) Correlation analysis of RAD51 and its auxiliary protein RAD51AP1 with drug sensitivity using the CTRP database. (B) Viability of si‐NC and si‐RAD51 HSC‐3 cells treated with 1–5 μg/mL cisplatin for 48h assessed by CCK‐8 assay. (C) Heatmap of correlations between RAD51 and 14 genes related to chemotherapy resistance pathways. (D) FANCD2, FANCA, FANCB, FANCC, FANCI and BLM mRNA expression in HSC‐3 cells post‐RAD51 knockdown. (E) RAD51‐related anticancer drugs from GDSC drug sensitivity. (F) RAD51‐related anticancer drugs from CTRP drug sensitivity. (ns, not significant, *p* ≥ 0.05; *p < 0.05; **p < 0.01 and ***p < 0.001).

Initially, we analysed the correlation between mRNA expression levels of RAD51 and its auxiliary protein RAD51AP1 with sensitivity to various anticancer drugs using the Cancer Therapeutics Response Portal (CTRP) database. RAD51 and RAD51AP1 showed significant negative correlations with sensitivity to multiple drugs (Figure 11A). Subsequently, HSC‐3 cells from si‐NC and si‐RAD51 groups were treated with cisplatin (1–5 μg/mL) for 48 h. CCK‐8 assays revealed significantly reduced viability in si‐RAD51 cells, indicating that RAD51 knockdown may impair chemoresistance (Figure 11B). Spearman correlation analysis revealed a significant positive correlation (*p* < 0.05) between RAD51 expression and genes within multiple chemotherapy resistance‐associated pathways, including the Fanconi anaemia pathway, homologous recombination and platinum drug resistance (Figure 11C). Given that the most pronounced decrease in viability occurred in si‐RAD51 cells treated with 2 μg/mL cisplatin, we assessed the mRNA expression of selected genes in this pathway. qRT‐PCR analysis demonstrated that RAD51 knockdown significantly reduced mRNA levels of FANCD2, FANCA, FANCB and FANCC (*p* < 0.05) Figure 11D. Although no significant effect was observed on BLM or FANCI mRNA expression, collectively, these results, integrated with our preceding experimental data, indicate that RAD51 deficiency suppresses chemotherapy resistance in the OSCC HSC‐3 cell line. Finally, leveraging the Genomics of Drug Sensitivity in Cancer (GDSC) (Figure 11E and Table S9) and CTRP databases (Figure 11F and Table S10), we identified compounds whose sensitivity positively correlated with RAD51 expression, suggesting their potential utility for targeting RAD51 in OSCC therapy.

## 5 Discussion

RAD51 is a highly conserved recombinase that plays a central role in homologous recombination repair (HRR)—a critical pathway for the precise repair of DNA double‐strand breaks (DSBs) [7]. As a key mediator of genomic stability, RAD51 facilitates the search for homologous DNA sequences and mediates strand exchange during HRR, thereby ensuring faithful DNA repair and replication fork restart [49]. Dysregulation of RAD51 is closely associated with tumourigenesis in multiple cancers, including breast [15], hepatocellular [8] and pancreatic carcinomas [11], with its overexpression frequently correlating with aggressive tumour behaviour, therapeutic resistance and poor prognosis. The functional integrity of RAD51‐mediated HRR is intrinsically linked to resistance against DNA‐damaging agents, such as platinum‐based chemotherapeutics and PARP inhibitors (PARPis) [50], underscoring its clinical relevance as a potential therapeutic target. To comprehensively investigate RAD51's multifaceted roles in OSCC, we conducted systematic multiomics bioinformatic analyses and experimental validations.

Our findings demonstrate that RAD51 is consistently overexpressed across pan‐cancer analyses and exhibits particularly pronounced upregulation in OSCC, where it shows high diagnostic power (AUC = 0.956 and 95% CI: 0.934–0.977). Elevated RAD51 expression is significantly associated with advanced tumour stage, poorer clinical outcomes and reduced treatment response. We further validated the upregulation of RAD51 at both the mRNA and protein levels using three independent datasets from the Gene Expression Omnibus (GEO) and immunohistochemical evidence from the Human Protein Atlas (HPA). The observed variation in overexpression levels across datasets is attributable to technical and cohort differences, yet the consistent directional change significantly bolsters our conclusion. Multiple prognostic models corroborated the association between high RAD51 expression and adverse survival in patients with OSCC, underscoring its critical role in disease progression. Functional enrichment analyses (GO/KEGG/GSEA) of RAD51‐coexpressed genes implicated its involvement in core oncogenic processes, such as DNA damage repair, cell cycle regulation and therapy resistance. These results provide a molecular framework for understanding RAD51's contribution to OSCC pathogenesis and highlight its potential as a promising therapeutic target for OSCC intervention. Single‐cell sequencing data from CancerSEA revealed a significant positive correlation between RAD51 expression and multiple malignant phenotypes, including cell proliferation, cell cycle progression, DNA damage repair and invasion, across diverse cancer types. This reinforces RAD51's role in driving aggressive tumour behaviour in OSCC. Analysis via the TISCH2 database indicated predominant expression of RAD51 in Tprolif and malignant cell clusters, suggesting its involvement in promoting OSCC progression through regulation of malignant phenotypes and remodelling of the tumour microenvironment (TME). These insights prompted additional immune infiltration analyses and the design of functional cellular assays for validation.

Immune infiltration analysis showed RAD51' significant negative correlations with 16 out of 24 immune cell subtypes (*p* < 0.05) and TME signatures (ImmuneScore: *R* = −0.180; StromalScore: *R* = −0.250 and ESTIMATEScore: *R* = −0.232, all *p* < 0.001). Previous studies indicate that inhibiting RAD51‐mediated DNA repair in small cell lung cancer causes DNA damage accumulation, activating the cGAS‐STING pathway to promote CD8^+^ T‐cell infiltration and enhance PD‐1/PD‐L1 checkpoint inhibitor efficacy [51]. In ovarian cancer, GRB2 maintains replication fork stability by inhibiting RAD51 ATPase activity, preventing cytosolic DNA release that activates cGAS‐STING; GRB2 deficiency allows PARP inhibitors (PARPis) to induce DNA fragment release and enhance antitumour immunity [52]. In pancreatic ductal adenocarcinoma, TSPAN7 inhibits RAD51AP1‐mediated DSB repair, activating cGAS‐STING signalling to increase CD8^+^ T‐cell infiltration and improve chemoimmunotherapy efficacy [53]. These findings suggest RAD51's dual role in tumour immunity: promoting cancer cell DNA repair/survival versus potentially enhancing immune recognition upon inhibition. Immune checkpoints critically regulate immune balance and tumours exploit them to evade surveillance [54]. RAD51 positively correlated with immunomodulatory genes in OSCC including TNFRSF18, CD40, LGALS9 and CD276. CD40 (TNFRSF member) activation promotes APC maturation but chronic signalling may induce tolerance [55]. LGALS9 inhibits T‐cell function via TIM‐3 binding [56]. CD276 (B7 family coinhibitor) suppresses NK/T‐cell activity [57]. TNFRSF18 enhances Treg immunosuppression [58]. High RAD51 expression may thus reduce PD‐1/PD‐L1 inhibitor efficacy, suggesting combinatorial targeting of RAD51 with checkpoints (e.g., CD276/LGALS9) could improve response. These correlations may form novel biomarker panels for predicting immunotherapy benefit or OSCC aggressiveness. In summary, targeting RAD51 or its pathways may improve cancer immunotherapy.

Next, to further elucidate RAD51's functional roles, we employed the HSC‐3 cell line—representing an aggressive tongue squamous cell carcinoma subtype of OSCC with particularly poor prognosis and unique therapeutic challenges. Consistent with RAD51's established oncogenic functions in other malignancies, our functional assays demonstrated that RAD51 depletion in HSC‐3 cells induced multiple phenotypic alterations: significantly inhibited cell proliferation, impaired migratory and invasive capacities, increased intracellular ROS accumulation and chemotherapy resistance. This aligns with reported effects of RAD51 knockdown in other cancers—suppressing proliferation and promoting ROS accumulation in pancreatic cancer [11], inhibiting proliferation/migration/invasion in cervical squamous carcinoma [12] and impeding proliferation/migration/invasion while enhancing apoptosis/DNA damage in liver cancer [8]. Furthermore, Spearman correlation analysis demonstrated a significant positive correlation (*p* < 0.05) between RAD51 expression and genes within multiple chemotherapy resistance‐associated pathways, including the Fanconi anaemia (FA) pathway, homologous recombination (HR) and platinum drug resistance. Activation or overexpression of the FA pathway can help cancer cells overcome or circumvent the DNA‐damaging effects induced by anticancer agents during treatment [59]. Conversely, impaired homologous recombination repair (HRR) leads to the accumulation of DNA damage, diminishing the cytotoxicity of chemotherapeutic drugs and adversely affecting patient prognosis [60]. Furthermore, cisplatin‐treated HSC‐3 cells subjected to RAD51 knockdown exhibited significantly reduced cell viability and diminished mRNA expression levels of key chemotherapy resistance‐related genes, including FANCD2, FANCA, FANCB and FANCC. Building upon our integrated experimental and bioinformatic evidence, future investigations could leverage deep learning frameworks to further explore RAD51's regulatory network in OSCC. By employing advanced deep learning models capable of integrating multiomics data [61, 62], researchers could systematically elucidate RAD51's global mechanisms in transcriptional regulation, immune microenvironment remodelling and chemotherapy resistance. Such analytical approaches would provide new perspectives for interpreting RAD51's noncanonical functions in OSCC and potentially offer theoretical foundations for developing combination therapies targeting RAD51‐high subtypes.

In conclusion, our integrated multiomics and experimental analysis establishes a foundational framework in which RAD51 acts as a central oncogenic driver in OSCC, promoting tumour progression through regulation of cell proliferation, invasion, chemoresistance and immune evasion. Although this study provides a comprehensive map of RAD51‐associated functions, it also defines specific avenues for immediate translation. The planned mechanistic dissection—using cisplatin‐treated transcriptomics, functional rescue assays and in vivo immune profiling—will be critical to translate the prognostic and therapeutic potential of RAD51 identified here into targeted strategies for OSCC treatment.

## Author Contributions

**Yuanxin Shi:** conceptualisation, data curation, methodology, software, validation, visualization, writing – original draft. **Xie Li:** conceptualisation, validation, visualization, writing – original draft. **Yueyue Wang:** data curation, software, writing – review and editing. **Bin Chen:** conceptualisation, data curation, funding acquisition, supervision, writing – review and editing. **Guohui Bai:** conceptualisation, data curation, funding acquisition, methodology, project administration, resources, supervision, writing – review and editing.

## Funding

This work was supported by the Guizhou Province Science and Technology Program (No. Qianke Heji‐ZK [2024] General 279), Key Discipline Construction Project of Higher Education Institutions in Guizhou Province (No.: Qianxuewei Hezi ZDKX (2017) 5), Talent Base Construction Project for the R&D of Medical Biomaterials in Guizhou Province and Zunyi City (No.: Qianrenling Fa (2018) 3, Zunwei (2019) 69) and Guizhou Province Key Laboratory Platform Project for Oral Disease Research of Ordinary Higher Education Institutions (Project No.: QianJiaoJiNian (2022) 025).

## Ethics Statement

The authors have nothing to report.

## Conflicts of Interest

The authors declare no conflicts of interest.

## Supporting information


**Figure S1:** Single‐cell RAD51 expression landscape in OSCC across three independent datasets (GSE139324, GSE172577 and GSE103322).

**Table S1:** Statistical analysis of RAD51 expression across pan‐cancer groups.

**Table S2:** Statistical analysis of pairwise comparisons of RAD51 expression across pan‐cancer groups.

**Table S3:** Baseline characteristics and RAD51 expression in the TCGA‐OSCC cohort.

**Table S4:** Pan‐cancer single‐cell analysis of RAD51‐related genes and malignant phenotypes.

**Table S5:** Spearman correlation analysis between RAD51 and other molecules in TCGA‐OSCC.

**Table S6:** GO enrichment analysis of the top 300 genes coexpressed with RAD51 in TCGA‐OSCC.

**Table S7:** KEGG enrichment analysis of the top 300 genes coexpressed with RAD51 in TCGA‐OSCC.

**Table S8:** Gene set enrichment analysis (GSEA) by RAD51 expression in TCGA‐OSCC.

**Table S9:** Correlation between RAD51 expression and anticancer compound sensitivity across human cancers (GDSC database).

**Table S10:** Correlation between RAD51 expression and anticancer compound sensitivity across human cancers (CTRP database).

## Data Availability

The data analysed during the current study are available in public repositories as specified in the Methods section. This study did not generate any new raw omics data.
