# Supplementary figures and images for: Multi‐Omics Analysis and Experimental Validation Identify RAD51 as a Key Biomarker in OSCC
Source: IET Syst Biol. 2025 Dec 5;19(1):e70048. doi: 10.1049/syb2.70048 (PMC12680491; doi:10.1049/syb2.70048)

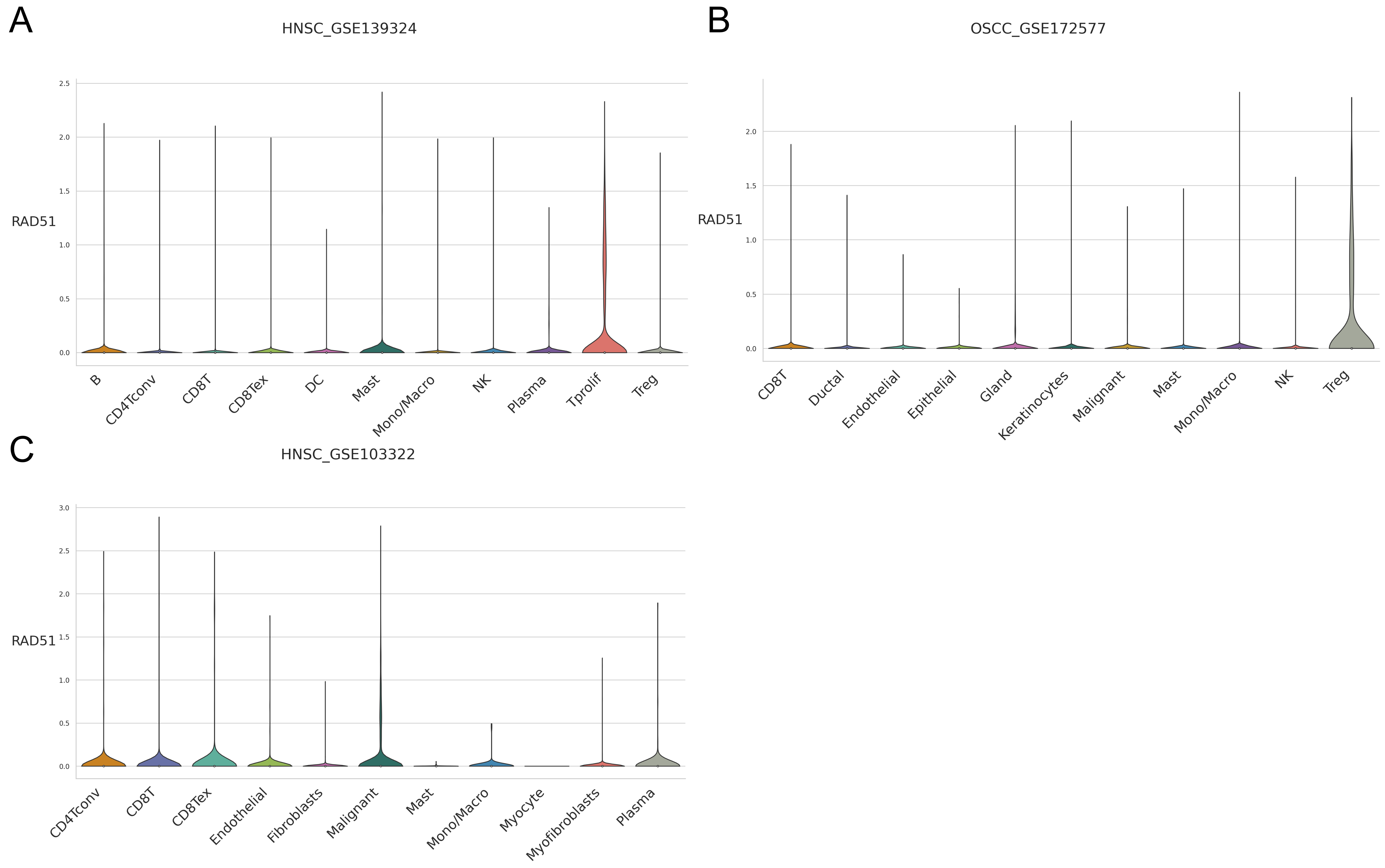

Supplement: Supplementary file 1 — Figure S1: Single‐cell RAD51 expression landscape in OSCC across three independent datasets (GSE139324, GSE172577 and GSE103322). [file SYB2-19-e70048-s005.tif]
